# Composition and physicochemical properties of outer mucilage from seeds of Arabidopsis natural accessions

**DOI:** 10.1093/aobpla/plz031

**Published:** 2019-06-28

**Authors:** Damien Poulain, Lucy Botran, Helen M North, Marie-Christine Ralet

**Affiliations:** 1 INRA, UR 1268 Biopolymères Interactions Assemblages, INRA, Nantes, France; 2 Institut Jean-Pierre Bourgin, INRA, AgroParisTech, CNRS, Université Paris-Saclay, Versailles Cedex, France

**Keywords:** *Arabidopsis thaliana*, intrinsic viscosity, molar mass, pectin, rhamnogalacturonan I, seed mucilage

## Abstract

*Arabidopsis thaliana* (Arabidopsis) seeds are myxospermous and release two layers of mucilage on imbibition. The outer layer can be extracted with water facilitating the analysis of its major constituent, polysaccharides. The composition and properties of outer mucilage have been determined for 306 natural accessions and six control genotypes to generate a data set comprising six traits measured in four biological replicates for each. Future exploitation of this data is possible in a range of analyses and should yield information concerning genetic diversity, underlying genetic factors and the biological function of mucilage as an adaptive trait.

## Teaser

The function of the viscous hydrogel that is formed around the seeds of many species on imbibition is currently unclear. This mucilage can either remain tightly adhered to the seed or leach into the surrounding soil, which is the case for the outer mucilage of Arabidopsis. We have generated a data set of outer mucilage traits for over 300 Arabidopsis accessions that can be exploited for examining its variation in relation to the environmental factors that vary with their geographical origin or with genome polymorphisms.

## Introduction

Seed mucilage is a hydrogel that is formed of polymers, mainly polysaccharides, released from seed coat epidermal cells on imbibition. This characteristic is termed myxospermy and is found in many plant species, including *Arabidopsis thaliana* (Arabidopsis) where mucilage is formed of two layers. Many roles have been proposed for seed mucilage, but to date no clear function has emerged in Arabidopsis, and as the two layers have different compositions and structure it is likely that they fulfil distinct functions (Macquet *et al.* 2007). While Arabidopsis is widely known as a model plant for genetic studies, it is also a weed whose natural habitat ranges through much of the northern hemisphere. Large numbers of natural Arabidopsis accessions have been collected from the wild and phenotypic variation for a number of traits has been described (reviewed in Weigel 2012). Natural selection infers that variation for traits should either improve fitness or be neutral, as those that create disadvantageous effects will be eliminated. The study of natural variation for a given trait can thus provide useful information about its physiological role through associations with population structure or geographical location.

In order to gain insight into the potential role(s) for outer mucilage the description of the variation observed within the Arabidopsis species is required. We have generated a data set for a panel of 306 natural Arabidopsis accessions, covering a variety of geographical locations and habitats, as well as six control genotypes. Specifically, this data set describes six traits for polysaccharides extracted from the outer layer of mucilage, which can be easily extracted from seeds with water. The composition of outer mucilage from the reference accession Col-0 was previously shown to be mainly unbranched rhamnogalacturonan I and this pectin is formed of a repeating disaccharide, [→2)-α-L-Rha-(1→4)-α-D-GalA-(1→]n. The analysis of GalA and neutral sugar contents thus provides information about the amount and composition of outer mucilage. The four remaining traits are related to the macromolecular properties of the polysaccharide polymers. The molar mass at peak maximum (Mp), intrinsic viscosity (IV), hydrodynamic radius (Rh) and radius of gyration (Rg) provide information about the conformation of the polymers. This information is not limited to individual values, but also the level of variation between values and the degree of correlation between different macromolecular parameters. Together these indicate the size of polymers and the volume they occupy as well as the level of heterogeneity for these characteristics within the population of polymers.

**Figure 1.**
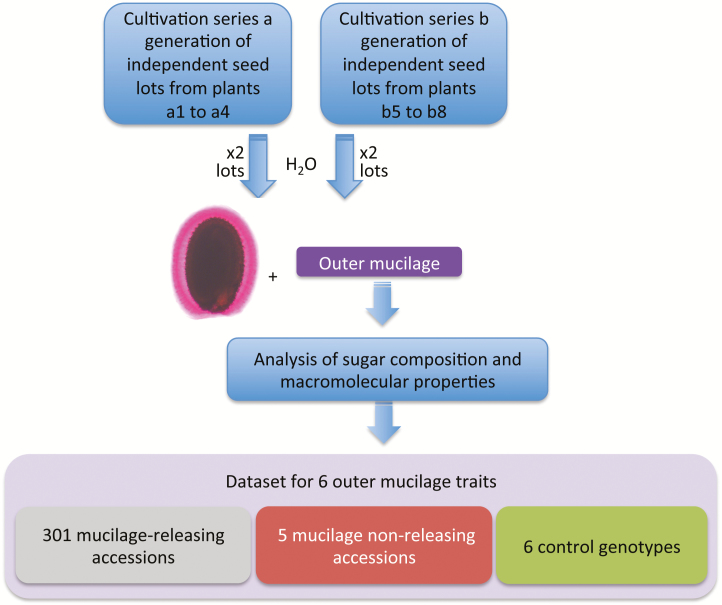
Schematic representation of the production of the data set for outer mucilage traits from seeds of Arabidopsis accessions and control genotypes. Outer mucilage was extracted with water from four seed lots generated from independent plants that had been produced at two different times corresponding to series a or b. Analyses of the sugar composition and macromolecular properties of outer mucilage extracts generated data for six traits for 306 accessions and six controls. The genotypes examined can be classed in three types depicted by colour blocks that correspond to shading used in the data set: 301 accessions that release mucilage, five accessions that do not release mucilage and the controls wild-type Col-0; *myb61*; *cesa5-1*; wild-type Col-2; *cesa5*^*mum3-1*^; *mum5-1*.

**Figure 2.**
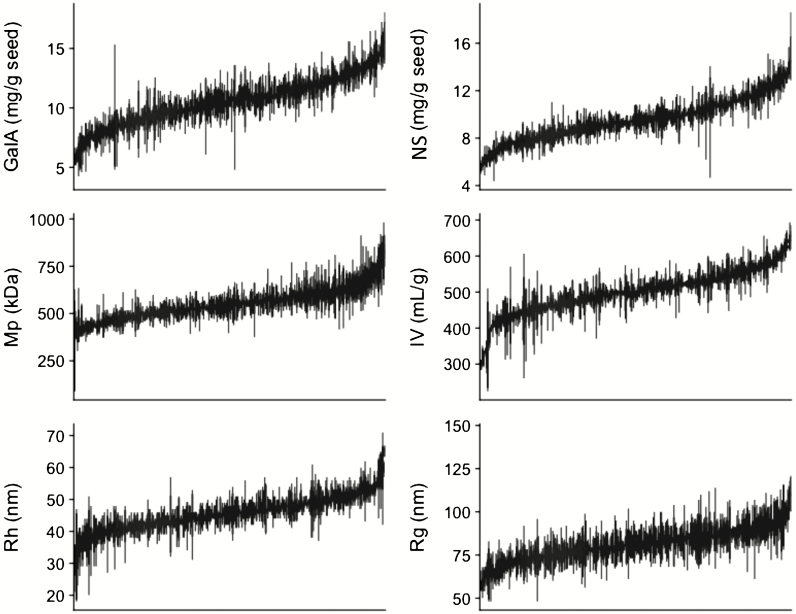
Ordered box plots of data for each outer seed mucilage variable sorted by accession with lowest to highest median value. GalA, galacturonic acid contents; NS, neutral sugar contents; Mp, molar mass at peak maximum; IV, intrinsic viscosity; Rh, hydrodynamic radius; Rg, radius of gyration.

The different steps of the data set production are summarized in Fig. 1. For each genotype four biologically independent seed lots were examined and the six traits were determined from water-extracted mucilage obtained from each seed lot. Five accessions known to produce seeds that do not release mucilage, and the six control genotypes whose outer mucilage has previously been characterized, represented internal quality controls; the extraction and analysis of samples were carried out blind to the expected phenotypes for these genotypes. Data values obtained for all control genotypes were coherent with previous results, thus indicating the reliability of the complete data set. The data values for each accession are summarized in box plots in Fig. 2, ordered by increasing median value, allowing the range of values obtained for each of the six variables to be observed.

This data set of outer mucilage traits provides a starting point for future analyses describing the genetic variation for these traits and the identification of accessions harbouring gene variants with strong effects. While the latter can be used to identify genes that contribute to the adaptive function of the traits through genetic linkage analysis using co-segregation, the data set as a whole could also provide valuable insights through association studies with other available data sets for genome sequence, phenotypes or geolocalization. The latter could highlight potential adaptations of mucilage traits to specific ecological or geographical environments.

## Data

The data set is available at the following link (https://doi.org/10.15454/Z4JM4F).

## Methods

### Plant material and growth conditions

The natural accessions were obtained from the Versailles Arabidopsis Stock Center (http://publiclines.inra.fr/naturalAccession/index) and are listed by their Versailles identification number in a four-digit format (i.e. 0001 for accession AV1). Available co-ordinates for the site of collection are indicated and classed for their precision using a grey to white colour scale indicating most to least reliable, respectively. Seed lots represent four independent biological repeats produced from different plants. These were produced from plants grown in a chamber with 65 % relative humidity and 170 µmol m^−2^ s^−1^ during a 16-h photoperiod at 21 °C and 8-h dark at 18 °C. Plants were grown in compost (Tref substrates) following a randomized sowing plan in two independent series of plants grown and harvested together, with four plants of each genotype per series. Series a were grown from December 2008 to June 2009 and series b from July to December 2009 using the same growth chamber. Seed lots were assigned sample codes a1 to a4 and b5 to b8 corresponding to seed lots harvested from independent plants from series a or b, respectively. For each accession two seed lots from each series were used for subsequent analyses. Control genotypes *cesa5-1* (SALK_125535), *myb61*, (SALK_106556) in Col-0 background and *cesa5*^*mum3-1*^ and *mum5-1* in the Col-2 background were obtained in previous studies (Western *et al.* 2001; Desprez *et al.* 2007; Saez-Aguayo *et al.* 2014). The code for control genotypes is wild-type Col-0, 4000; *myb61*, 1000; *cesa5-1*, 2000; wild-type Col-2, 7000; *cesa5*^*mum3-1*^, 5000; *mum5-1*, 6000. Four seed lots were also obtained for controls from independent plants grown as described above; their data are colour-coded in green. A red colour code indicates data for known accessions affected in mucilage release, Shahdara, Neo-3, Neo-6 and Sus-1 (Macquet *et al.* 2007; Saez-Aguayo *et al.* 2014) or for Dja-5, which has the same haplotype as Dja-1, which shows delayed and incomplete mucilage release in water (Simon *et al.* 2012; Saez-Aguayo *et al.* 2014).

### Mucilage extraction and analysis

Mucilage was extracted from 100 mg of seed with 2 mL of water by head-to-tail mixing for 3 h at 20 °C. Extracts were then centrifuged at 8000 g for 3 min and filtered through a disposable glass microfiber filter (13 mm diameter, 2.7 µm pore size). A 200 µL aliquot was diluted 10-fold in water and analysed (San-System, Skalar) by the automated *m*-hydroxybiphenyl method and the automated orcinol method to determine uronic acid and neutral sugar contents, respectively (Thibault 1979; Tollier and Robin 1979). The remaining extract was heated to 100 °C for 5 min and stored at −20 °C. Just prior to high-performance size exclusion chromatography (HPSEC) analysis, this extract was thawed, heated again to 100 °C and filtered through a disposable polyvinylidene fluoride (PVDF) filter (13 mm diameter, 0.45 µm pore size, Whatman). HPSEC analysis was performed at room temperature with a Shodex OH Pak SB-G-6B precolumn and a Shodex OH Pak SB805 HQ column. Elution was carried out with 50 mM sodium nitrate at 0.7 mL min^−1^. The following detectors were used, a differential refractometer (Viscotek VE 3580 RI detector) and a dual light scattering detector combined with a differential pressure viscometer (Viscotek 270 Dual detector). Calibration was performed daily with a pullulan standard for triple calibration (Malvern) with a narrow molar mass distribution (Mw 145618D, Mn 139180D, intrinsic viscosity 54 mL g^−1^).

## Sources of Funding

This work was funded by the ANR program (grant numbers ANR-08-BLAN-0061 and ANR-14-CE19-0001-01).

## Conflict of Interest

None declared.

## Contributions by the Authors

M.-C.R. and H.M.N. conceived and supervised experiments, analyzed the data and wrote and revised the article; D.P. and L.B. performed experiments.

## References

[CIT0001] Desprez T, Juraniec M, Crowell EF, Jouy H, Pochylova Z, Parcy F, Hofte H, Gonneau M, Vernhettes S 2007 Organization of cellulose synthase complexes involved in primary cell wall synthesis in *Arabidopsis thaliana*. Proceedings of the National Academy of Sciences of the United States of America 104:15572–15577. 17878303 10.1073/pnas.0706569104 PMC2000492

[CIT0002] Macquet A, Ralet MC, Loudet O, Kronenberger J, Mouille G, Marion-Poll A, North HM 2007 A naturally occurring mutation in an *Arabidopsis* accession affects a beta-D-galactosidase that increases the hydrophilic potential of rhamnogalacturonan I in seed mucilage. The Plant Cell 19:3990–4006. 18165330 10.1105/tpc.107.050179 PMC2217647

[CIT0003] Saez-Aguayo S, Rondeau-Mouro C, Macquet A, Kronholm I, Ralet MC, Berger A, Sallé C, Poulain D, Granier F, Botran L, Loudet O, de Meaux J, Marion-Poll A, North HM 2014 Local evolution of seed flotation in *Arabidopsis*. PLoS Genetics 10:e1004221. 24625826 10.1371/journal.pgen.1004221 PMC3953066

[CIT0004] Simon M, Simon A, Martins F, Botran L, Tisné S, Granier F, Loudet O, Camilleri C 2012 DNA fingerprinting and new tools for fine-scale discrimination of *Arabidopsis thaliana* accessions. The Plant Journal 69:1094–1101. 22077701 10.1111/j.1365-313X.2011.04852.x

[CIT0005] Thibault J-F 1979 Automatisation du dosage des substances pectiques par la méthode au métahydroxydiphényle. Lebensm Wissenschaft und Technologie 12:247–251.

[CIT0006] Tollier MT, Robin JP 1979 Adaptation de la méthode à l’orcinol sulfurique au dosage automatique des glucides neutres totaux: conditions d’application aux extraits d’origine végétale. Annales de Technologie Agricole 28:1–15.

[CIT0007] Weigel D 2012 Natural variation in *Arabidopsis*: from molecular genetics to ecological genomics. Plant Physiology 158:2–22. 22147517 10.1104/pp.111.189845 PMC3252104

[CIT0008] Western TL, Burn J, Tan WL, Skinner DJ, Martin-McCaffrey L, Moffatt BA, Haughn GW 2001 Isolation and characterization of mutants defective in seed coat mucilage secretory cell development in *Arabidopsis*. Plant Physiology 127:998–1011. 11706181 PMC129270

